# Phase I Study of Safety and Pharmacokinetics of RO7297089, an Anti-BCMA/CD16a Bispecific Antibody, in Patients with Relapsed, Refractory Multiple Myeloma

**DOI:** 10.1007/s44228-022-00023-5

**Published:** 2023-01-19

**Authors:** Torben Plesner, Simon J. Harrison, Hang Quach, Cindy Lee, Adam Bryant, Annette Vangsted, Jane Estell, Michel Delforge, Fritz Offner, Patrick Twomey, Voleak Choeurng, Junyi Li, Robert Hendricks, Shannon M. Ruppert, Teiko Sumiyoshi, Karen Miller, Eunpi Cho, Fredrik Schjesvold

**Affiliations:** 1 Vejle Hospital, University of Southern Denmark, Vejle, Denmark; 2 Peter MacCallum Cancer Center, Royal Melbourne Hospital, Melbourne and Sir Peter MacCallum Dept of Oncology University of Melbourne, Parkville, Australia; 3 St. Vincent’s Hospital Melbourne, University of Melbourne, Melbourne, Australia; 4 Royal Adelaide Hospital, Adelaide, Australia; 5 Liverpool Hospital, Sydney, Australia; 6 Department of Hematology, Rigshopitalet, Copenhagen, Denmark; 7 Concord Repatriation General Hospital, Concord, Australia; 8 University Hospitals Leuven, Louvain, Belgium; 9 Ghent University, Ghent, Belgium; 10 Genentech, Inc., South San Francisco, CA USA; 11 Oslo Myeloma Center, Department of Hematology, Oslo University Hospital, Oslo, Norway; 12 KG Jebsen Center for B Cell Malignancies, University of Oslo, Oslo, Norway

**Keywords:** RO7297089, Multiple myeloma, BCMA, CD16a, Clinical trial

## Abstract

**Introduction:**

This phase 1 trial assessed the safety, pharmacokinetics, and preliminary antitumor activity of RO7297089, an anti-BCMA/CD16a bispecific antibody.

**Methods:**

RO7297089 was administered weekly by intravenous infusion to patients with relapsed/refractory multiple myeloma. The starting dose was 60 mg in this dose-escalation study utilizing a modified continual reassessment method with overdose control model.

**Results:**

Overall, 27 patients were treated at doses between 60 and 1850 mg. The maximally administered dose was 1850 mg due to excipients in the formulation that did not allow for higher doses to be used. The maximum tolerated dose was not reached. The most common adverse events irrespective of grade and relationship to the drug were anemia, infusion-related reaction, and thrombocytopenia. Most common treatment-related grade ≥ 3 toxicities were ALT/AST increase and reduced lymphocyte count. Pharmacokinetic studies suggested non-linear pharmacokinetics and target-mediated drug disposition, with a trend of approaching linear pharmacokinetics at doses of 1080 mg and higher. Partial response was observed in two patients (7%), minimal response in two patients (7%), and stable disease in 14 patients (52%).

**Conclusions:**

RO7297089 was well tolerated at doses up to 1850 mg, and the efficacy data supported activity of RO7297089 in multiple myeloma. Combination with other agents may further enhance its potential as an innate immune cell engager in multiple myeloma.

**Trial Registration:**

ClinicalTrials.gov: NCT04434469; Registered June 16, 2020; https://www.clinicaltrials.gov/ct2/show/NCT04434469.

## Introduction

Multiple myeloma (MM) is a cancer of malignant plasma cells. Despite advances in treatment, the estimated median survival is 8–10 years for standard-risk myeloma and 2–3 years for high-risk disease, despite receipt of an autologous stem cell transplant [1]. The successful use of monoclonal antibodies in MM (e.g., daratumumab and elotuzumab) provides evidence that antibody-dependent cell-mediated cytotoxicity mediated by natural killer (NK) cells may be an important therapeutic mechanism for MM.

RO7297089 is a bispecific, tetravalent antibody targeting B-cell maturation antigen (BCMA) and CD16a. BCMA is exclusively expressed on plasmablasts and plasma cells. It is over-expressed in malignant plasma cells, and its expression level is reported to be associated with disease progression [2–4]. CD16a, also known as FcɣRIIIa, is expressed on mast cells, macrophages, NK cells, and monocyte subsets as a transmembrane receptor [5, 6]. Engagement of BCMA and CD16a by RO7297089 results in the formation of an immunological synapse to mediate target-specific killing of BCMA-positive cells by CD16a-positive immune effector cells, such as NK cells and macrophages. In vitro models have demonstrated potential advantages of the CD16a construct over the native FCɣR, including high affinity monovalent binding to both CD16A allotypes 158V and 158F, no detectable binding to primary human granulocytes, and retained NK cell binding in the presence of competing IgG. These models have also demonstrated the antiproliferative effects of RO7297089 via the in vitro lysis of myeloma cells through CD16A-expressing immune effectors [7]. Furthermore, the in vitro cytokine release assays and in vivo safety data in nonhuman primates suggested a favorable safety profile [7]. Based on these preclinical data, we performed a clinical study of RO7297089 in patients with relapsed/refractory MM (RRMM).

## Methods

### Study Design

This was a phase 1, multicenter, open-label, dose-escalation study of RO7297089 administered as a single agent to patients with RRMM (ClinicalTrials.gov: NCT04434469). The primary objectives were to evaluate the safety and tolerability of RO7297089 and to determine the maximum tolerated dose (MTD) and dose-limiting toxicities (DLTs). Secondary objectives included assessments for pharmacokinetics (PK), efficacy, immunogenicity of RO7297089, and to identify the recommended phase-2 dose at or below the MTD. RO7297089 was administered at 60–1850 mg by intravenous infusion on Days 1 and 8 of each 14-days cycle (Arm A). A minimum of three patients were enrolled at each dose level, and the maximal dose escalation increment recommended by the model was threefold. The study utilized a modified continual reassessment method of escalation with overdose control model to determine the MTD, with a plan for cohort expansion at the recommended phase-2 dose. Relevant demographic, adverse event, laboratory, dose administration, and available PK data were reviewed prior to dose-escalation decisions, which were made by an Internal Safety Committee, in consultation with the study investigators. Patients were monitored for DLTs after the first infusion during the DLT assessment window (Cycle 1, Days 1–14 [C1D1-14]). Intrapatient dose escalation was permitted to the highest cleared dose level, provided that the patient completed at least two cycles of RO7297089 at the originally assigned dose.

To mitigate the risk of infusion-related reactions (IRRs), the protocol was amended in September 2020 to require glucocorticoid, antihistamine, and antipyretic medications prior to the first dose of RO7297089. To accommodate long infusion times of larger doses, the protocol was additionally amended in November 2020 to add an option for up to three dose-escalation arms (Arms A, B, and C) that differed in the RO7297089 Cycle 1 administration schedule in a given dose cohort. Arm B (allowing the first dose in Cycle 1 to be divided over C1D1 and C1D2) was opened for the 1080 mg and 1850 mg cohorts; Arm C (administering 60 mg on C1D1 and full target dose on Day 8) was not opened at any dose level. Per investigator discretion, some patients received additional IRR prophylaxis including corticosteroids on the day prior to the first infusion, corticosteroids for one or two days following the first infusion, or montelukast prior to the first infusion.

This study was conducted in accordance with Good Clinical Practice guidelines and the Declaration of Helsinki. Written informed consent was obtained from all patients prior to enrollment, in agreement with approved protocols from ethics committees at all study sites.

### Patients

Eligible patients were 18 years of age or older with histologic documentation of RRMM which had progressed on or following prior therapy, and for which no standard therapy existed. Other inclusion criteria were: Eastern Cooperative Oncology Group performance status of 0 or 1; availability and willingness to provide an adequate archival tumor sample; absolute neutrophil count ≥ 1000/μL, hemoglobin ≥ 8 g/dL, and platelet count ≥ 50,000/μL; total bilirubin ≤ 1.5 × the upper limit of normal (ULN); aspartate transaminase (AST) and alanine transaminase (ALT) levels ≤ 3 × the ULN; creatinine clearance ≥ 30 mL/minute; measurable disease as defined by serum monoclonal protein ≥ 0.5 g/dL, urine monoclonal protein ≥ 200 mg/24 h, or serum-free light chains (SFLC); involved SFLC ≥ 10 mg/dL and an abnormal SFLC ratio. Patients who did not meet criteria for hematologic function because of MM-related cytopenias were permitted to enroll. Exclusion criteria were: systemic antitumor therapy within 4 weeks or 5 half-lives prior to Day 1; active infection; current grade > 1 toxicity (except alopecia, anorexia, and fatigue) from prior therapy; autologous stem cell transplantation within 100 days prior to Day 1; CAR-T therapy within 90 days prior to Day 1; history of severe allergic or anaphylactic reactions to monoclonal antibody therapy; untreated or active central nervous system involvement with MM; evidence of significant uncontrolled concomitant diseases including cardiac, nervous system, pulmonary, renal, hepatic, endocrine, or gastrointestinal disorders. Prior therapy with other BCMA-targeting agents were permitted. Evidence of BCMA expression was not required.

### Safety

Adverse events (AEs) were graded according to the NCI Common Terminology Criteria for Adverse Events v5.0, except for cytokine-release syndrome, which was graded according to the Modified Cytokine-Release Syndrome Grading System [8].

The following AEs considered by the investigator to be related to RO7297089 and occurring during Days 1–14 of Cycle 1 in dose-escalation cohorts were considered a DLT: any Grade 4 or 5 AE, Grade 3 febrile neutropenia lasting > 3 days, Grade 3 elevations of serum hepatic transaminases lasting > 7 days, and Grade 3 non-hematologic events with the following exceptions: Grade 3 nausea, vomiting, or diarrhea that improves to Grade ≤ 2 with standard‑of-care therapy in ≤ 3 days, Grade 3 fatigue that improves to Grade ≤ 2 within 7 days, Grade 3 infection that resolves within 7 days to Grade ≤ 2 and does not require intensive care unit transfer, fractures of any grade at site of lytic bone disease, and Grade 3 IRRs. Grade 3 laboratory abnormalities that were asymptomatic, resolved to Grade ≤ 1 or baseline levels within 7 days, and were considered by the investigator not to be clinically significant were also not considered DLT.

### Pharmacokinetics and Immunogenicity

PK samples of RO7297089 were collected at pre-specified intervals described in the protocol (e.g. pre-dose, end of infusion [EOI], 2–4 h, 24 h, 48 h and 168 h after EOI for first dose, and pre-dose and EOI sample for subsequent doses) to capture the concentration–time curve, and enable characterization of key PK parameters (e.g. AUC, *C*_max_, *C*_min_, *t*_1/2_). Serum concentrations of total RO7297089 were analyzed by a validated total ELISA assay (unbound and bound to target in serum), which requires the presence of both BCMA and CD16a arms; the lower limit of quantitation was 100 ng/mL. PK parameters for RO7297089 in C1D1 dose were estimated by standard non-compartmental analysis using WinNonlin 5.2.1 software. At subsequent cycles, the peak and trough (pre-infusion) concentrations were summarized using descriptive statistics.

The samples to detect antidrug antibody (ADA) were collected at pre-dose of every cycle for the first three cycles, and every other cycle for Cycles 2, 4, 8 to characterize the pre-existing ADA and post-treatment ADA profiles. Detection of ADAs to RO7297089 in human serum was performed using a validated bridging ELISA method. Using a mouse monoclonal anti-BCMA antibody as a surrogate positive control, the relative sensitivity was determined to be 62.0 ng/mL.

### Biomarkers

Blood samples, bone marrow aspirate, and bone marrow core biopsy samples were collected at pre-specified intervals described in the protocol. Soluble BCMA and soluble CD16a levels were measured at baseline and at post-treatment timepoints. Immune cell populations were analyzed by flow cytometry.

### Antitumor Activity

Response was assessed by investigators using the 2016 International Myeloma Working Group (IMWG) response criteria [9] at odd number cycles until disease progression, death, start of new anti-cancer therapy, withdrawal of consent for study participation, or end of the study, whichever occurred first.

### Statistical Analysis

The planned enrollment for the dose-escalation cohorts was 30–50 patients. All analyses included patients who received any amount of RO7297089 according to the assigned dose level. Safety data were assessed through summaries of AEs, laboratory test results, and vital signs. Response outcomes and progression endpoints were assessed for all patients and by indication using the IMWG criteria.

## Results

### Patient Population

From 8 July 2020 to 16 February 2022, a total of 27 patients were enrolled in the dose-escalation cohorts across 5 dose levels (*n* = 3 at 60 mg, *n* = 5 at 180 mg, *n* = 4 at 360 mg, *n* = 12 at 1080 mg and *n* = 3 at 1850 mg). Seven patients underwent intra-patient dose escalation. Patient demographics and baseline characteristics are summarized in Table 1. Patients had received a median of seven prior lines of therapy (range 2–12).

**Table 1:** Baseline demographics and disease characteristics

	All patients (*N* = 27)
Age, years, median (range)	62 (41–76)
Gender, female	8 (30%)
Race	
Asian	1 (4%)
White	24 (89%)
Unknown	2 (7%)
Baseline ECOG status	
0	15 (56%)
1	12 (44%)
Prior lines of therapy, median (range)	7 (2–12)
Prior BCMA-targeted therapy	5 (24%)
High risk cytogenetics*	14 (52%)
Extramedullary disease at screening	10 (37%)
Prior proteasome inhibitors	27 (100%)
Prior immunomodulatory drugs	27 (100%)
Prior anti-CD38 antibody	26 (96%)
Triple-class refractory	20 (74%)
Penta-drug refractory	13 (48%)

*BCMA* B-cell maturation antigen, *ECOG* Eastern Cooperative Oncology Group

*Defined by presence of del(17p), *t*(4;14), *t*(14;16), *t*(14;20), gain 1q or p53 mutation

### Safety

The median number of weekly doses of RO7297089 administered was 8.0 (range 3–53); median duration of safety follow-up was 6 months (range 1.0–16.1). Of the 27 patients, 25 (92.6%) experienced at least one AE regardless of their relationship to the study drug (Table 2). A total of 14 (52%) patients experienced AEs related to RO7297089, the most common of which were IRRs (*n* = 10), ALT increase (*n* = 4), AST increase (*n* = 4) and C-reactive protein increase (*n* = 3). Increases in ALT and AST occurred in the context of IRR during the first cycle and were transient. A total of 17 (63%) patients experienced Grade ≥ 3 AEs regardless of attribution to study drug, the most common of which were anemia (*n* = 8) and platelet count decrease (*n* = 6). Grade ≥ 3 AEs considered related to study drug were ALT and AST increases, and lymphocyte count decrease (all *n* = 1). The Grade 3 AST and ALT elevations were transient following IRR, and happened with both Cycle 1 infusions in 1 patient, being resolved within 3–5 days on both occasions. No DLTs were reported. AEs did not result in treatment discontinuation for any patients. Five deaths were reported in the AE follow-up period, all due to disease progression.

**Table 2:** All adverse events occurring in ≥ 10% of patients regardless of attribution by initially assigned dose

Adverse event	RO7297089 First assigned dose											
	60 mg (*n* = 3)		180 mg (*n* = 5)		360 mg (*n* = 4)		1080 mg (*n* = 12)		1850 mg (*n* = 3)		All patients (*N* = 27)	
	Any grade	Grade 3–4	Any grade	Grade 3–4	Any grade	Grade 3–4	Any grade	Grade 3–4	Any grade	Grade 3–4	Any grade	Grade 3–4
Hematologic AE												
Anemia	1 (33%)	1 (33%)	3 (60%)	3 (60%)	3 (75%)	1 (25%)	3 (25%)	3 (25%)	0	0	10 (37%)	8 (30%)
Platelet count decreased*	1 (33%)	1 (33%)	3 (60%)	2 (40%)	2 (50%)	2 (50%)	2 (17%)	1 (8%)	0	0	8 (30%)	6 (22%)
Non-hematologic AE												
Infusion-related reaction	1 (33%)	0	3 (60%)	0	3 (75%)	0	3 (25%)	0	0	0	10 (37%)	0
Back pain	1 (33%)	0	1 (20%)	0	0	0	4 (33%)	1 (8%)	1 (33%)	0	7 (26%)	1 (4%)
ALT increased	0	0	3 (60%)	1 (20%)	0	0	2 (17%)	0	0	0	5 (19%)	1 (4%)
AST increased	0	0	2 (40%)	1 (20%)	0	0	2 (17%)	0	0	0	4 (15%)	1 (4%)
Diarrhea	1 (33%)	0	1 (20%)	0	0	0	2 (17%)	0	0	0	4 (15%)	0
Fatigue	1 (33%)	0	0	0	1 (25%)	1 (25%)	2 (17%)	0	0	0	4 (15%)	1 (4%)
Arthralgia	0	0	0	0	0	0	3 (25%)	0	0	0	3 (11%)	0
CRP increased	1 (33%)	0	1 (20%)	0	0	0	1 (8%)	0	0	0	3 (11%)	0
Dyspnea	0	0	2 (40%)	0	0	0	1 (8%)	0	0	0	3 (11%)	0
Headache	0	0	0	0	0	0	2 (17%)	0	1 (33%)	0	3 (11%)	0
Nausea	0	0	0	0	0	0	2 (17%)	0	1 (33%)	0	3 (11%)	0
Pyrexia	1 (33%)	0	1 (20%)	0	0	0	1 (8%)	0	0	0	4 (15%)	0

*AE* adverse event, *ALT* alanine transaminase, *AST* aspartate transaminase, *CRP* C-reactive protein

*Platelet count decreased includes the terms thrombocytopenia and platelet count decreased

Thirteen IRRs (1 Grade 1, 12 Grade 2) were observed in 10 patients (37%; 1 patient at 60 mg, 3 at 180 mg, 3 at 360 mg, 2 at 1080 mg in Arm A, 1 patient at 1080 mg in Arm B, and no patients at 1850 mg in Arm B). Pre-medications were made mandatory after the first five patients were treated (3 at 60 mg and 2 at 180 mg). IRRs were most common in Cycle 1 (12 events in 10 patients) and uncommon in subsequent cycles (1 patient). Symptoms of IRR in > 1 patient included fever, rigors/chills, and hypertension. Of the 12 patients treated in Arm B (first dose administered over 2 days), there was only 1 with an IRR. No patients experienced an IRR following a new higher dose with intra-patient escalation. Infusion rates could be increased with later doses and corticosteroid pre-medication could be tapered/discontinued without recurrence of IRR. Cytokine release syndrome was reported in two patients (7.4%): one experienced two events of Grade 1 CRS and the other one event of Grade 2 CRS. Both patients responded to supportive care. No immune effector cell-associated neurotoxicity syndrome (ICANS) was reported.

The MTD was not reached in this study and the maximum administered dose (MAD) was 1850 mg. Additional escalation was not performed due to excipients in the formulation, including sucrose at concentrations that led to capping the dose at 1850 mg.

### Pharmacokinetics and Immunogenicity

**Fig. 1:**
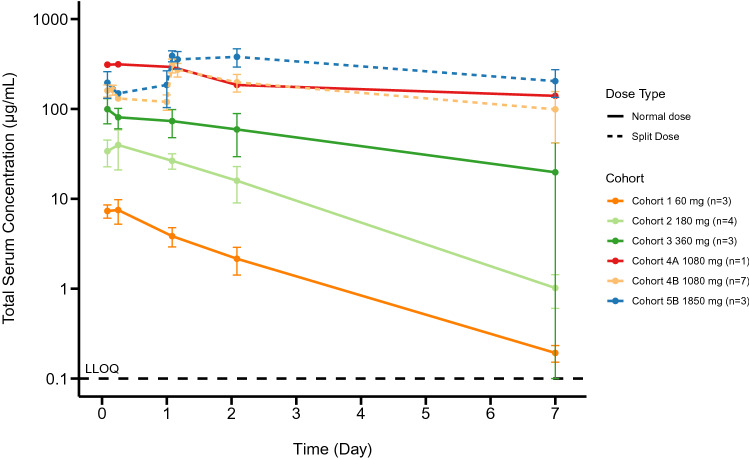
Mean (±SD) RO7297089 serum concentration–time profiles following the first dose in patients administered a flat dose (Cohorts 1-4A) 60–1080 mg or a split dose (Cohorts 4B & 5B) 1080 and 1850 mg QW. Dashed line at the bottom indicates the lower limit of quantitation. PK data from 6 patients were excluded from the plot due to not receiving the full dose, missed sample, and an obvious outlier with *C*_trough_ concentration higher than concentration at the end of infusion

PK assessments (Fig. 1) demonstrated a more than dose-proportional increase in the exposure of RO7297089 with increasing dose levels from 60 to 1080 mg, suggesting non-linear PK and target-mediated drug disposition and a trend for approaching linear PK at doses 1080 mg and higher. The apparent half-life was about 1.3–6.7 days, which is supportive of weekly dosing.

The baseline prevalence of anti-RO729789 antibodies was 1 of 27 (3.7%) for patients with a baseline ADA sample. Two of 27 (7.4%) patients were positive for treatment-emergent ADAs, which did not appear to impact the apparent PK exposure. Patient 150,004 (Cohort 2) tested positive for ADAs at C2D1 with a titer of 1.99 (titer units) and at C3D1 with a titer of 1.93 (titer units). The patient subsequently tested negative at C4D1, C6D1 and end of treatment. Patient 180,001 (Cohort 4b) tested positive for ADAs at C3D1 with a titer of 2.41 (titer units), and negative at all other time points tested C2D1, C4D1, and end of treatment.

### Antitumor Activity

**Fig. 2:**
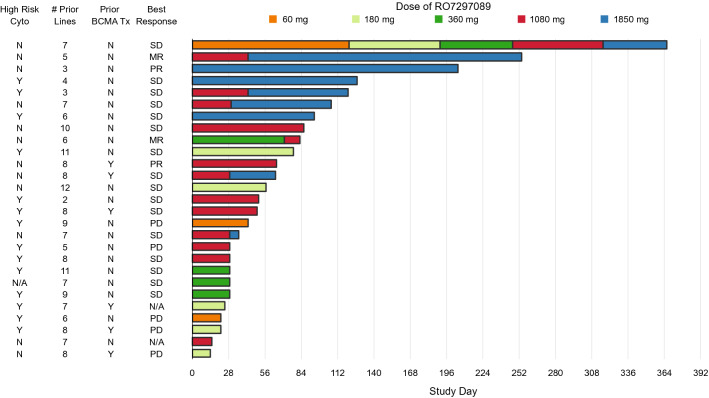
Time on treatment and summary of responses. Intra-patient dose escalation was permitted to the highest cleared dose level, provided that the patient completed at least 2 cycles of RO7297089 at the originally assigned dose. Seven patients underwent intra-patient dose escalation and are detailed in the bar graph. *MR* minimal response, *N/A* assessment not done, *PD* progressive disease, *PR* partial response, *SD* stable disease, *tx* treatment. For patients with prior anti-BCMA treatment, all treatments consisted of belantamab mafodotin

Of the 27 response-evaluable patients, 2 (7%) experienced partial response (PR) per IMWG criteria (1 patient each at 1080 and 1850 mg) (Fig. 2). Two patients (7%) had minimal response (MR) as their best response at 360 mg (1/5 patients) and 1080 mg (1/12 patients). Sixteen patients (59%) had stable disease as their best response at 60 mg (1/3 patients), 180 mg (2/5 patients), 360 mg (3/4 patients), 1080 mg (8/12 patients), and 1850 mg (2/3 patients). One patient who started at 60 mg and gradually escalated to 1850 mg was on treatment for 27 cycles prior to discontinuation for progressive disease. This prolonged duration of disease stability was notable for this patient, who was penta-exposed and had seven lines of therapy prior to enrolling on this study. There was one patient who was naive to daratumumab; this patient achieved a PR and stayed on treatment for 16 cycles prior to discontinuation for progressive disease. Three of 27 patients remained on study treatment for at least 6 months. Information on the potential association of high-risk cytogenetics and efficacy are limited due to small patient numbers; notably, patients achieving PR or MR did not have high-risk cytogenetics.

### Biomarker Assessments

While evidence of BCMA expression was not required for enrollment, it was assessed and detected on bone marrow aspirate samples by flow cytometry for all enrolled patients, regardless of prior BCMA-targeted therapy.

Peripheral blood immune cell subsets were assessed by flow cytometry with a focus on NK cells and monocytes, since these cells are known to express CD16a. Sixteen patients (59%) had baseline NK counts within the normal range of 100–650 cells/uL. The other 11 patients had NK counts below the normal range. Seventeen patients (63%) had monocyte counts within the normal range; the other 12 patients had monocyte counts below normal range (< 200 cells/µL). There was no correlation between baseline NK cell or monocyte numbers and clinical activity of RO729089. There was also no apparent association between prior therapy and baseline NK and monocyte counts. Eleven of 27 patients received a CD38 antibody in the treatment regimen just prior to study entry; of these, 3 had NK cell counts below and 8 within the normal range.

Levels of soluble BCMA and CD16a were also assessed at baseline and post-treatment. The median soluble BCMA level at baseline was 151 ng/mL (range: 8.01–2980 ng/mL), similar to levels previously reported for patients with RRMM. Both soluble targets, given their size, are expected to have a shorter half-life in circulation than RO7297089. An increase in levels of both soluble targets immediately after the first dose was observed, suggesting stabilization of soluble targets by RO7297089. At doses of 1080 mg and 1850 mg, the level of RO7297089 was > twofold higher than the levels of soluble BCMA and soluble CD16a in the majority of patients, suggesting these doses would be high enough to overcome a potential soluble antigen sink in the peripheral blood.

IL-6 levels were assessed at baseline and post-treatment. IL-6 elevations of ≥ 100 pg/mL after the first dose were observed in 4 of 6 patients in the first two cohorts prior to implementation of mandatory corticosteroid premedication and slower infusion rates. These elevations were associated with IRR. Following the amendment, only 2 of 21 patients demonstrated IL-6 elevations, suggesting that the increase in IL-6 can be mitigated by adjustment of the infusion rate and pre-medication.

## Discussion

Results from this phase 1 study demonstrated that RO7297089 has an acceptable safety profile when administered by intravenous infusion every week in patients with RRMM. On the basis of the protocol-specified criteria, an MTD for single-agent RO7297089 was not reached, and the MAD was 1850 mg. The most common RO7297089-related AE across dose levels was IRR, predominantly associated with the first infusion. A slower infusion rate and pre-medication with a corticosteroid, antihistamine, and antipyretic mitigated these events.

PR was observed in two patients (at 1080 mg and 1850 mg) and MR in two patients (360 mg and 1080 mg), and three patients (11%) received study treatment for ≥ 6 months (all three patients either assigned or escalated to 1850 mg). The single-agent efficacy seen in this study was not as robust as what has been reported with BCMA-targeted T-cell engagers [10]. One possible explanation for the moderate clinical activity of RO7297089 is its relatively long half-life, which may down-tune the function of NK cells over time. Tonic activation of NK cells is known to induce a state of hypo-responsiveness that may limit the ability of NK cells to respond to repeat infusions [11]. Another possibility is that additional optimization of dose or development of combination strategies may be required to advance this agent in clinical development.

The non-linear PK observed at lower doses of RO7297089 likely reflects the impact on drug clearance, due to target-mediated drug disposition. The soluble targets (e.g. soluble BCMA and CD16a) also likely acted as soluble sinks which, in addition to having a potential impact on RO7297089 PK, may also be affecting the level of free drug that would be available to link NK cells with tumor cells. At doses of 1080 mg and higher, the levels of RO7297089 were likely high enough to overcome the potential soluble antigen sink in the peripheral blood. The RO7297089 exposure level and the sink effect of soluble antigens at the target site (bone marrow) needs further assessment. Doses above 1850 mg could not be tested due to excipient content in the formulation limiting further dose escalation.

## Conclusions

In conclusion, RO7297089 demonstrated an encouraging safety and PK profiles, as well as evidence of antitumor activity of an innate immune cell engager in MM. Single-agent dose expansion was not pursued due to limited single-agent activity at the tested doses. Additional modifications to RO7297089 may allow improved formulation to permit higher doses or subcutaneous delivery, to potentially improve patient convenience and safety. Combining RO7297089 with agents that enhance NK cell activity could be interesting avenues to explore, which could potentially deepen or broaden the responses seen with RO7297089 monotherapy.

## Data Availability

For eligible studies, qualified researchers may request access to individual patient level clinical data through a data request platform. At the time of writing this request platform is Vivli: https://vivli.org/ourmember/roche/. For up to date details on Roche's Global Policy on the Sharing of Clinical Information and how to request access to related clinical study documents, see here: https://go.roche.com/data_sharing. Anonymized records for individual patients across more than one data source external to Roche cannot, and should not, be linked due to a potential increase in risk of patient re-identification.

## Abbreviations

AE: Adverse event
ADA: Antidrug antibody
ALT: Alanine transaminase
AST: Aspartate transaminase
BCMA: B-cell maturation agent
C#D#: Cycle # Day #
DLT: Dose-limiting toxicities
EOI: End of infusion
IMWG: International Myeloma Working Group
IRR: Infusion-related reaction
MAD: Maximum administered dose
MM: Multiple myeloma
MR: Minimal response
MTD: Maximum tolerated dose
NK: Natural killer
PK: Pharmacokinetics
PR: Partial response
RRMM: Relapsed/refractory multiple myeloma
SFLC: Serum free light chains
ULN: Upper limit of normal

## References

[1] Mikhael JR, Dingli D, Roy V, Reeder CB, Buadi FK, Hayman SR, Dispenzieri A, Fonseca R, Sher T, Kyle RA, Lin Y, Russell SJ, Kumar S, Bergsagel PL, Zeldenrust SR, Leung N, Drake MT, Kapoor P, Ansell SM, Witzig TE, Lust JA, Dalton RJ, Gertz MA, Stewart AK, Rajkumar SV, Chanan-Khan A, Lacy MQ, Mayo C (2013). Management of newly diagnosed symptomatic multiple myeloma: updated Mayo Stratification of Myeloma and Risk-Adapted Therapy (mSMART) consensus guidelines 2013. Mayo Clin Proc.

[2] Lee L, Bounds D, Paterson J, Herledan G, Sully K, Seestaller-Wehr LM, Fieles WE, Tunstead J, McCahon L, Germaschewski FM, Mayes PA, Craigen JL, Rodriguez-Justo M, Yong KL (2016). Evaluation of B cell maturation antigen as a target for antibody drug conjugate mediated cytotoxicity in multiple myeloma. Br J Haematol.

[3] Seckinger A, Delgado JA, Moser S, Moreno L, Neuber B, Grab A, Lipp S, Merino J, Prosper F, Emde M, Delon C, Latzko M, Gianotti R, Luoend R, Murr R, Hosse RJ, Harnisch LJ, Bacac M, Fauti T, Klein C, Zabaleta A, Hillengass J, Cavalcanti-Adam EA, Ho AD, Hundemer M, San Miguel JF, Strein K, Umana P, Hose D, Paiva B, Vu MD (2017). Target expression, generation, preclinical activity, and pharmacokinetics of the BCMA-T cell bispecific antibody EM801 for multiple myeloma treatment. Cancer Cell.

[4] Tai YT, Anderson KC (2019). B cell maturation antigen (BCMA)-based immunotherapy for multiple myeloma. Expert Opin Biol Ther.

[5] Ravetch JV, Perussia B (1989). Alternative membrane forms of Fc gamma RIII(CD16) on human natural killer cells and neutrophils. Cell type-specific expression of two genes that differ in single nucleotide substitutions. J Exp Med.

[6] Wong KL, Tai JJ, Wong WC, Han H, Sem X, Yeap WH, Kourilsky P, Wong SC (2011). Gene expression profiling reveals the defining features of the classical, intermediate, and nonclassical human monocyte subsets. Blood.

[7] Kakiuchi-Kiyota S, Ross T, Wallweber HA, Kiefer JR, Schutten MM, Adedeji AO, Cai H, Hendricks R, Cohen S, Myneni S, Liu L, Fullerton A, Corr N, Yu L, de Almeida ND, Zhong S, Leong SR, Li J, Nakamura R, Sumiyoshi T, Li J, Ovacik AM, Zheng B, Dillon M, Spiess C, Wingert S, Rajkovic E, Ellwanger K, Reusch U, Polson AG (2022). A BCMA/CD16A bispecific innate cell engager for the treatment of multiple myeloma. Leukemia.

[8] Lee DW, Santomasso BD, Locke FL, Ghobadi A, Turtle CJ, Brudno JN, Maus MV, Park JH, Mead E, Pavletic S, Go WY, Eldjerou L, Gardner RA, Frey N, Curran KJ, Peggs K, Pasquini M, DiPersio JF, van den Brink MRM, Komanduri KV, Grupp SA, Neelapu SS (2019). ASTCT consensus grading for cytokine release syndrome and neurologic toxicity associated with immune effector cells. Biol Blood Marrow Transplant.

[9] Kumar S, Paiva B, Anderson KC, Durie B, Landgren O, Moreau P, Munshi N, Lonial S, Blade J, Mateos MV, Dimopoulos M, Kastritis E, Boccadoro M, Orlowski R, Goldschmidt H, Spencer A, Hou J, Chng WJ, Usmani SZ, Zamagni E, Shimizu K, Jagannath S, Johnsen HE, Terpos E, Reiman A, Kyle RA, Sonneveld P, Richardson PG, McCarthy P, Ludwig H, Chen W, Cavo M, Harousseau JL, Lentzsch S, Hillengass J, Palumbo A, Orfao A, Rajkumar SV, Miguel JS, Avet-Loiseau H (2016). International Myeloma Working Group consensus criteria for response and minimal residual disease assessment in multiple myeloma. Lancet Oncol.

[10] Usmani SZ, Garfall AL, van de Donk N, Nahi H, San-Miguel JF, Oriol A, Rosinol L, Chari A, Bhutani M, Karlin L, Benboubker L, Pei L, Verona R, Girgis S, Stephenson T, Elsayed Y, Infante J, Goldberg JD, Banerjee A, Mateos MV, Krishnan A (2021). Teclistamab, a B-cell maturation antigen x CD3 bispecific antibody, in patients with relapsed or refractory multiple myeloma (MajesTEC-1): a multicentre, open-label, single-arm, phase 1 study. Lancet.

[11] Goodridge JP, Onfelt B, Malmberg KJ (2015). Newtonian cell interactions shape natural killer cell education. Immunol Rev.

